# Cervically Embedded Fragment of an Intrauterine Device in a Patient with a Bicornuate Uterus: A Case Report and Review of Current Management Strategies

**DOI:** 10.7759/cureus.40938

**Published:** 2023-06-25

**Authors:** Alexandra C Skoczek, Jacqueline Sylvester

**Affiliations:** 1 Medicine, Edward Via College of Osteopathic Medicine - Auburn, Huntsville, USA; 2 Obstetrics and Gynecology, Crestwood Medical Center, Huntsville, USA

**Keywords:** clinical case report, surgical hysteroscopy, intrauterine device malfunction, intrauterine devices, bicornuate uterus

## Abstract

Intrauterine devices (IUDs) have become one of the most frequently used forms of long-acting reversible contraception (LARC) in women of childbearing age. While complications are generally considered to be minimal, they can occur during the insertion, during use, or upon removal. Uterine anomalies, such as a bicornuate uterus, can increase the risk of complications during all stages. Here, we describe a case of a patient with a bicornuate uterus who had a levonorgestrel IUD in place for five years before she experienced a dislodging of the IUD, fragmentation upon attempted removal, and ultimately required a hysteroscopy to remove an embedded fragment from the endocervical canal. Due to the limited reporting on fragmented IUDs, further studies will be required to assess the optimal management. While symptomatic patients should have the fragment removed, asymptomatic patients should have their individual history and desire for future pregnancy weighed against the risk and benefits of treatment.

## Introduction

Intrauterine devices (IUDs) are a form of long-acting reversible contraception (LARC) that can effectively prevent pregnancy between three and 10 years [1]. Currently marketed IUDs in the United States include the non-hormonal copper 380 mm^2^ IUD and the hormonal levonorgestrel (13.5 mg, 19.5 mg, and 52 mg) IUD [1]. IUDs are a highly effective method of LARC with 0.6% of copper IUD users and 0.1-0.3% of levonorgestrel IUD users experiencing pregnancy within the first year. This is up to 20 times more effective than combined oral contraceptive pills [1,2]. Due to their high efficacy and long duration, IUDs are becoming one of the most widely used forms of reversible contraception. According to the National Survey of Family Growth, between 2017 and 2019, 65.3% of biological women aged 15-49 in the United States were using some form of contraception [3]. 8.4% of women reported using an IUD, with a higher usage seen in women aged 20-29 [3]. The usage of IUDs has steadily increased since the early 2000s with only 1.3% of women reporting IUD use in 2002 [4]. 

While IUDs have relatively low complication rates, complications can include expulsion, uterine perforation, ectopic pregnancy, and pelvic inflammatory disease [1,5]. Patients with uterine cavity anomalies, untreated cervical cancer, and active pelvic infections have a contraindication to IUD insertion due to the increased risk of complications [1]. A bicornuate uterus is a structural uterine anomaly due to a failure of fusion of the bilateral Mullerian ducts. Patients have a partial duplication of the uterus with a single cervix and vagina. The endometrial cavities are separate but communicate with one another [6]. In patients with an IUD having a bicornuate uterus increases the risk of expulsion, pregnancy, bleeding, perforation, and pain [7]. In the following case, we describe a case of an embedded fragment of an IUD in a patient with a bicornuate uterus who subsequently underwent a hysteroscopy for fragment removal. 

## Case presentation

A 44-year-old, G5P0141, female with a past medical history of recurrent miscarriages and a bicornuate uterus presented to the emergency department with intense suprapubic abdominal pain. The patient stated that she was diagnosed with a bicornuate uterus after three miscarriages and one late second-trimester stillbirth. After her diagnosis, she was successfully able to have a child that was born at 37 weeks via vaginal delivery after an unknown procedure for her bicornuate uterus. Following her successful pregnancy and delivery five years ago, she had a levonorgestrel IUD placed by her obstetrician. For the past five years, she had no complications with her IUD and did not experience regular menstrual periods. 

Five days prior to her presentation in the emergency department, she states she was seen in a different emergency department out of state after she experienced intense lower abdominal cramping and heavy uterine bleeding. An ultrasound performed at that emergency visit diagnosed her with a dislodged IUD, and after receiving pain medications and fluids, she was discharged home and instructed to follow up with her physician for IUD removal. The patient was unable to follow up with the obstetrician who had placed the IUD due to relocation and instead followed up with her family provider for removal. The patient was seen by her family provider two days prior to this emergency department visit for IUD removal. During the attempted removal, the IUD fragmented into multiple pieces and was complicated by “excessive blood loss.” The family provider was able to successfully remove the retrieval strings, stem, and one of the arms; however, was unable to locate and remove the second arm. The patient was reassured that the second arm would most likely be expelled by her uterus and left the office without plans for further follow-up.

**Figure 1:**
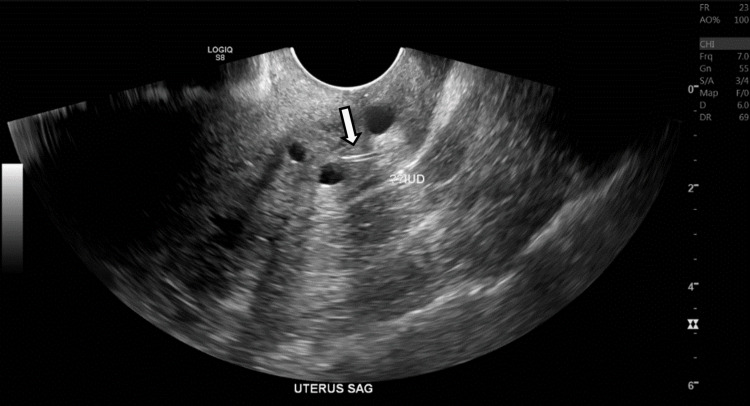
Transvaginal Ultrasound Showing Suspected IUD Fragment

The patient stated that a few hours prior to her arrival in the emergency department she began to experience intense suprapubic abdominal pain described as a stabbing in addition to nausea. She denied any other symptoms including vaginal bleeding, vomiting, fever, or urinary symptoms. On examination, the patient was in visible distress secondary to pain. Abdominal examination was remarkable for suprapubic abdominal tenderness and voluntary guarding. The rest of her examination was unremarkable. Complete metabolic panel and complete blood count values were within normal limits. A transvaginal ultrasound revealed a 1.1 cm linear echogenic structure with posterior shadowing embedded in the posterior wall of the cervix (Figure 1). This was suspected to be the fragment of the IUD arm. After reviewing the findings with the patient, a hysteroscopy was planned for that same afternoon. 

**Figure 2:**
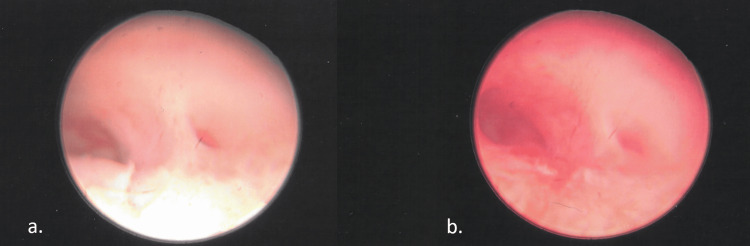
Hysteroscopic Findings of a Bicornuate Uterus Images *a* and *b* both taken during hysteroscopy show the separatory cleft between left and right uterine openings.

**Figure 3:**
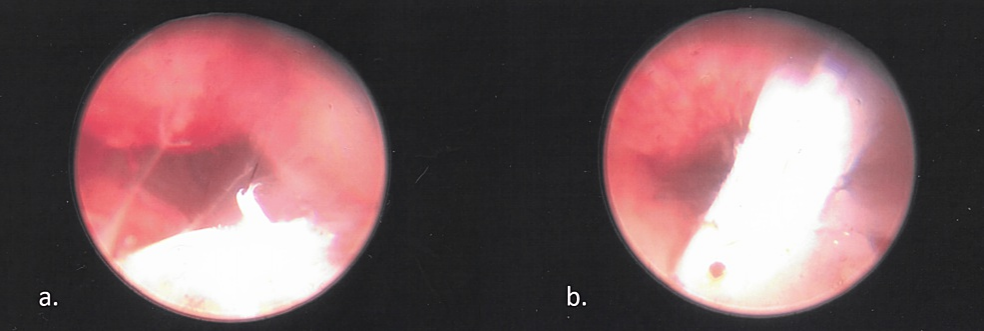
IUD Fragment Found on Hysteroscopy Images *a *and *b *show a small IUD fragment found in the mid cervix on hysteroscopy.

The patient was taken to the operating room and a hysteroscopy was performed under general anesthesia. Upon insertion of the hysteroscopy, the bicornuate uterus was noted (Figure 2). No fragment of the IUD could be identified. After gentle probing of the lower uterine segment to the mid cervix, a small IUD remnant was found (Figure 3). Multiple attempts were required to free portions of the fragment, broken into several pieces. In attempting to remove the fragmented piece one of the instruments broke and an additional instrument had to be brought in. After all the pieces had appeared to be removed the fragments were sent off to pathology and a sharp curettage was performed in the region. Hysteroscopy was performed again, and no additional pieces could be identified. Instrumentation was then removed, and the patient was given ceftriaxone for antibiotic prophylaxis and ketorolac for pain. Upon awakening from the procedure, the patient was stable and in minimal pain and was able to be discharged home. 

## Discussion

IUD complications vary in severity and can be heavily influenced by patient history (nulliparity, uterine abnormalities, age), IUD type (hormonal versus nonhormonal), as well as differences in childbirth (placement time following childbirth and delivery type) [5,8]. The majority of complications follow insertion and are present during IUD use. Of these, dysmenorrhea is the most common complication being seen in up to 25% of individuals, followed by ectopic pregnancy seen in 2.9% to 8.9% of individuals, and uterine perforation is seen in up to 1.3% of individuals [9]. Expulsion can occur between 2% and 10% following insertion in a non-postpartum patient; however, this varies greatly following delivery and by type of delivery [10]. A meta-analysis by Averbach et al. noted IUD expulsion in as high as 14.8% of patients following vaginal delivery with an expulsion rate of 27.4% in patients who had a levonorgestrel IUD placed immediately after delivery [8]. 

While most complications occur in the period following insertion, complications can occur during IUD removal. These complications include missing strings, incomplete removal, and fragmentation. Missing IUD strings can occur in a correctly positioned IUD where the strings have retracted or be indicative of a larger complication such as an IUD that has been expelled or perforated the uterus [10]. A prospective cohort of 350 individuals receiving an immediate post-placental copper IUD by Dewan et al. revealed that missing strings were more commonly seen in cesarean deliveries when compared to vaginal [11]. 

A far less studied and reported removal complication is incomplete removal with the fragmentation of the IUD. Food and Drug Administration Adverse Event Reporting System (FAERS) reported a breakage in 9.6% of copper IUD removals and 1.7% of hormonal IUD removals [12]. The exact etiology of fragmentation is unknown; however, it is theorized that fragmentation may be due to the structural breakdown of the device over time, contractile forces from the myometrium, or partial embedment of a portion of the IUD [10]. Currently, limited case reports have documented fragmentation following the removal of an IUD after being in place for multiple years [13-16]. While some cases report heavy menstrual bleeding and pain prior to removal, possibly signifying dislodging of the IUD, other cases report no complications prior to removal [15,16]. In accordance with FAERS, the majority of cases published on IUD fragmentation occur in copper IUD usage [12-16]. While minimally reported in the literature, having a Mullerian abnormality such as a bicornuate uterus further increases all complication rates both following insertion and upon removal.

A bicornuate uterus is a relatively rare diagnosis with a prevalence of 0.4% in the general population [17]. It is typically diagnosed on hysterosalpingography, ultrasound, or MRI and is most frequently diagnosed after a workup for recurrent miscarriage or infertility [17]. While having a bicornuate uterus significantly increases both pre- and post-gestational complications, it has also been seen to increase complications in patients with LARC. Multiple case studies have reported instances of pregnancy in patients with a bicornuate uterus where the pregnancy occurs in one horn with the IUD in the other [7,18,19]. Other commonly reported complications include perforation, spontaneous abortions, and abnormal uterine bleeding [7]. Due to known complications and limited studies on IUD as a LARC in women with uterine abnormalities, they are not currently recommended for contraception [1]. 

In this case, multiple factors could have led to the fragmentation of the IUD. The patient’s history of a bicornuate uterus predisposed her to all IUD complications. It is unknown which bicornuate horn the IUD was initially placed in. Hysteroscopy revealed one horn with a much narrower cavity than the other. If placed in the narrower horn there would be an increased likelihood of an embedment of a portion of the IUD leading to possible fragmentation upon removal. Due to limited documented cases of fragmentation, management is debated.

Of the cases reviewed, the most reliable detection of IUD fragment location includes ultrasound or hysteroscopy. X-ray imaging may be an inexpensive method to determine if fragmentation has occurred; however, it is not reliable in providing the location of the fragment or to determine if the fragment has become embedded in surrounding structures [13]. CT scans can also be employed if there is suspicion of perforation or intraabdominal migration of the IUD or IUD fragment but should not be used as the initial diagnostic imaging [13-15]. If the fragment of IUD has not penetrated the myometrium or endocervical canal, the use of manual vacuum aspiration (MVA) or IUD hook/narrow tip forceps under ultrasound guidance can be employed and may be readily available to practitioners in the office [15]. A sample of cases by Wilson et al. noted increased success with MVA with a lower risk of uterine perforation [15]. When the fragment has become deeply embedded in the myometrium or the endocervical canal, further methods may need to be employed for successful removal.

When embedded in the myometrium or endocervical canal, successful removal has been documented using hysteroscopy or hysterectomy, dependent on patient history [13-16]. Two cases of an embedded copper IUD within the uterine cavity required hysterectomy; however, in those cases the patients had a previous history of adenomyosis and abnormal bleeding combined with the wish to no longer have children [13]. Other cases, including our own, reported successful removal using hysteroscopy; however, in these cases the IUD, while embedded, was easily visualized and accessible [14-16]. While no studies have compared the success rate and complications of hysterectomy versus hysteroscopy, the patient’s gynecological history as well as the desire to bear children in the future should be taken into consideration. 

While an embedded fragment of an IUD may result in pain and bleeding, some patients may remain asymptomatic and fragmentation may only be noted after inspection of the IUD following removal [15]. In asymptomatic cases, the risks and benefits of removal must be considered. In postmenopausal women, the fragment may have a decreased risk of migration and perforation due to the lack of menstruation. Premenopausal patients, however, may experience expulsion of the fragment during menstruation or the fragment may become more accessible [15]. This may allow for elective removal. Patients who desire future pregnancy must be further counseled on the risks and benefits. A retained fragment may initiate the inflammatory response leading to an increased risk of adverse pregnancy outcomes such as infertility, spontaneous abortion, or preterm labor. However, removal of the fragment may result in uterine perforation requiring a hysterectomy, infection, and adhesions, all of which would affect future fertility [1,13,15]. Currently, no prospective studies have been conducted evaluating the long-term risk of a retained IUD fragment, thus each case should be individualized to the patient and their current clinical situation. In the presented case, the patient was experiencing significant pain due to the fragment, thus, the benefits of hysteroscopy outweighed the risk. In addition, the patient was aware of the complications, including the possible need for a hysterectomy, and no longer desired children should the need for a hysterectomy arise. 

## Conclusions

In summary, we present a case of a fragmented IUD in a patient with a bicornuate uterus who subsequently required a hysteroscopy for fragment removal. Her history of a bicornuate uterus may have increased her likelihood of IUD complications. While current management is controversial it is agreed that the management should be tailored to the individual case to prevent further complications.
